# CNOT6L regulates energy metabolism in the ovarian granulosa cells associated with polycystic ovary syndrome

**DOI:** 10.3389/fcell.2025.1607161

**Published:** 2025-05-19

**Authors:** Shan Han, Yanqiu Xie, Jiale Lv, Xuedong Sun, Yuhua Shi

**Affiliations:** ^1^ Guangdong Cardiovascular Institute, Guangdong Provincial People’s Hospital, Guangdong Academy of Medical Sciences, Guangzhou, China; ^2^ Department of Reproductive Medicine, Guangdong Provincial People’s Hospital (Guangdong Academy of Medical Sciences), Southern Medical University, Guangzhou, China; ^3^ Department of Reproductive Medicine, Nanfang Hospital, Southern Medical University, Guangzhou, China

**Keywords:** CNOT6L, polycystic ovary syndrome, poly(A) tail, granulosa cells, energy metabolism

## Abstract

The endocrine functions exerted by ovarian granulosa cells (GCs) are crucial factors in maintaining follicle development, as oocyte development relies on providing energy substrates and cytokines by ovarian granulosa cells. The mRNA deadenylase level of granulosa cells precisely regulates the follicular development processes. In this study, we detect the expression level of the deadenylase CNOT6L in polycystic ovarian syndrome (PCOS) patients’ granulosa cells and mouse models’ ovaries. The results found that the CNOT6L significantly upregulated in the ovarian granulosa cells of both PCOS patients and mouse models. Subsequently, we conducted the *Cnot6l-*overexpressed granulosa cells to explore the alterations by which CNOT6L regulates ovarian granulosa cell function. The overexpression of CNOT6L in granulosa cells significantly inhibited the glycolytic pathway, activated the mitochondrial oxidative phosphorylation pathway, led to a reduction in the generation of the intermediate product lactate, and resulted in impaired energy supply to the oocyte. Subsequently, we performed Full-length transcriptome sequencing on the granulosa cells and investigated the impact of mRNA poly(A) level differences on granulosa cell dysfunction in PCOS. This study offers new insights into the role of CNOT6L in regulating energy metabolism homeostasis and its involvement in follicular developmental disorders related to polycystic ovary syndrome.

## Introduction

Polycystic ovarian syndrome (PCOS) is the most prevalent gynecological endocrine disorder among women of reproductive age, with an estimated prevalence ranging from 6% to 20% (Yildiz et al., 2012; Escobar-Morreale, 2018). The pathophysiology of PCOS is complex, often resulting in anovulatory infertility and hyperandrogenism, which significantly impact the fertility of women of childbearing age. PCOS is also associated with severe metabolic syndrome, including type 2 diabetes mellitus (T2DM) and obesity (Yildiz et al., 2012; Neven et al., 2018). The prevalence of severe obesity among women with PCOS exceeds 25% (Yildiz et al., 2012; Neven et al., 2018; Kataoka et al., 2019). In clinical practice, ovulation induction therapy is the preferred treatment for infertility in PCOS patients, while *in vitro* fertilization and embryo transfer (IVF-ET) is the recommended fertility treatment for those with refractory PCOS (Joham et al., 2022). Previous studies have identified a high degree of heterogeneity among PCOS patients, with significantly lower outcomes in terms of the number of oocytes retrieved, oocyte maturity, normal fertilization rates, and the number of transferable high-quality embryos compared to patients with normal ovarian function. This condition places a significant strain on healthcare resources; therefore, it is crucial to investigate the mechanisms underlying follicle development disorder and anovulation in patients with polycystic ovary syndrome.

During oocyte maturation, ovarian granulosa cells, which are somatic cells encircling the oocyte, actively interact with the developing oocyte. Previous RNA-Seq analysis of ovarian granulosa cells associated with germinal vesicle (GV) and metaphase II (MII) stage oocytes in clinical patients demonstrated that genes targeted by follicle-stimulating hormone (FSH), including the steroidogenic acute regulatory protein (StAR), show elevated expression levels in MII stage GCs (Babayev and Duncan, 2022; Yerushalmi et al., 2014). The modifications in the expression of hormone-synthesizing genes responsive to gonadotropins within granulosa cells were observed to coincide with the oocyte development process. The findings indicate that the microenvironment of oocyte-granulosa cells is integral to the precise regulation of oocyte development (Li et al., 2021).

Post-transcriptional regulation of mRNA is a precise method for controlling gene expression. In most eukaryotes, the mRNA’s 3′ end is modified with Poly(A) tails, which are regulated and altered in length and composition within the cell. The metabolism of the dynamically changing mRNA poly(A) tail represents a crucial node in gene regulation, exerting a significant influence on protein translation, mRNA stability, and a range of physiological and pathological processes, including embryonic development. De-adenylation of mRNA is a key step in initiating transcription silencing. In eukaryotes, adenylates near the 3′ end are primarily removed by the CCR4-NOT protein complex. This complex serves as the principal non-specific deadenylase in eukaryotic cells and comprises seven core subunits, including exonuclease CCR4 (CNOT6 or CNOT6L). Recent studies have confirmed the involvement of CNOT6/6L and CNOT7 in the meiotic processes of oogenesis and spermatogenesis (Brachova et al., 2021; Dai et al., 2021). However, their specificity and function in other cell types require further investigation.

Research involving animal models has demonstrated that female *Cnot6l* ^−/−^ mice exhibit infertility or sterility. In the ovaries of *Cnot6/6l* ^*−/−*^ female mice, the quantities of primordial, primary, and secondary follicles were comparable to those observed in wild-type mice; however, the number of antral follicles was reduced significantly compared to control mice. This observation suggests that the deletion of CNOT6 and CNOT6L impairs the transition from secondary to antral follicles. Furthermore, although ovulation can be induced in these mice through superovulatory treatment, the oocytes display defective spindle assembly and encounter blocked embryonic development post-fertilization (Dai et al., 2021; Dai et al., 2022). This manifestation parallels the clinical characteristics observed in patients with polycystic ovary syndrome. Specifically, PCOS patients undergoing ovulation induction therapy for infertility experience a decline in oocyte quality, primarily evidenced by reduced cellular maturity, increased abnormal oocytes morphology, decreased fertilization rates, and a lower quality of embryos (Qiao and Feng, 2011; Xu et al., 2022). Consequently, there are fewer embryos available for transfer and diminished quality scores for transferred embryos. In a PCOS-like mouse model induced by prenatal exposure to anti-Mullerian hormone, an upregulation of deadenylase expression was noted in the PCOS-like group (Mimouni et al., 2021). These findings suggest that the abnormal expression of deadenylase genes may be a characteristic feature in the ovarian granulosa cells of individuals with polycystic ovary syndrome.

There is an increasing interest in the potential role of CNOT6L in metabolic regulation and disease. Analysis using the Comparative Toxicogenomics Database has indicated plausible associations between CNOT6L and metabolic disorders, obesity, and insulin sensitivity. Elevated expression of CNOT6L is notably observed in type 2 diabetes, where it contributes to diabetic complications, inflammation, and other physiological processes through the regulation of miRNA and related signaling pathways, ultimately resulting in a poor prognosis. Consequently, CNOT6L may represent a potential therapeutic target for type 2 diabetes (Zhang et al., 2024). Recent research has demonstrated that CNOT6L plays a significant role in the regulation of energy within peripheral tissues and organs. The inhibition of CNOT6L results in the stabilization of hepatic Gdf15 and Fgf21 mRNA, as well as serum protein levels, which in turn stimulates the hypothalamus to suppress appetite. This process also influences the liver and adipose tissues to enhance energy expenditure and promote lipid depletion (Katsumura et al., 2022). Furthermore, CNOT6L is implicated in the regulation of glucose transporter and energy transporter processes by modulating glycolysis, mitochondrial function, and cellular thermogenesis. These actions collectively impact energy conversion and accumulation in hepatocytes and adipocytes.

Follicular development and metabolic disorders are two critical manifestations associated with the onset and progression of polycystic ovary syndrome. Although several studies have confirmed the involvement of CNOT6L in oocyte development and the maintenance of energy metabolism homeostasis in hepatic tissue, the precise relationship between CNOT6L and PCOS remains unclear and warrants further investigation. Furthermore, granulosa cells serve as essential structures providing energy substrates and hormones for oocytes. However, research exploring the relationship between mRNA poly(A) tail deadenylation induced by CNOT6L in granulosa cells and the pathogenesis of PCOS is still lacking. Consequently, leveraging clinical data from PCOS patients and animal models, research focusing on the role of CNOT6L in modulating energy metabolism in the granulosa cells of individuals with PCOS holds substantial research value and scientific significance.

## Methods

### Human study

This study received approval from the Institutional Review Board of the Center for Reproductive Medicine at Guangdong Provincial Hospital (Approval Number: KY2024-473-01) and adhered to the principles outlined in the Declaration of Helsinki. Written informed consent was obtained from all participants. The study included a total of 27 patients diagnosed with polycystic ovary syndrome in the PCOS group, while 22 women who underwent *in vitro* fertilization due to male factor infertility or tubal factor infertility were included in the control group. All participants were under the age of 40. Both groups underwent IVF cycles between 2019 and 2021. The PCOS group met the inclusion criteria based on the modified Rotterdam criteria, whereas the control group was selected based on specific inclusion criteria, which included regular menstrual cycles, the absence of endocrine abnormalities, and confirmation of normal ovarian and uterine morphology through ultrasound or histological examination. Exclusion criteria for all participants included recurrent pregnancy loss, chromosomal abnormalities, diabetes mellitus, adenomyosis, endometriosis, and a history of malignancy. All women with basal hormone levels were included in this study, with their levels assessed on the third day of the menstrual cycle using chemiluminescence immunoassays. Blood samples were collected to measure Anti-Mullerian Hormone (AMH) levels, also utilizing chemiluminescence immunoassays. Body Mass Index (BMI) was calculated using the formula weight (kg)/height (m^2^). The waist-to-hip ratio, an index of body fat distribution, was determined by dividing waist circumference (cm) by hip circumference (cm). The antral follicle count for all subjects in the cohort study was measured on the assessment day via transvaginal ultrasound. Participants in each cohort underwent ovarian stimulation following standardized long-agonist protocols. In this protocol, pituitary function was suppressed using a gonadotropin-releasing hormone agonist (GnRHa) on day 21 of the preceding menstrual cycle, followed by the administration of recombinant Follicle-Stimulating Hormone (FSH) at doses ranging from 75 to 225 IU. In all protocols, urinary human chorionic gonadotropin (hCG) was administered once at least two dominant follicles reached a minimum diameter of 18–20 mm. Oocyte retrieval was performed 34–36 h after hCG injection through vaginal ultrasound-guided follicle puncture. During a single treatment cycle, the maturation process was assessed by determining the absolute number of mature oocytes and the percentage of mature oocytes collected. Additionally, follicular fluid was collected to isolate ovarian granulosa cells during the oocyte retrieval procedure.

### Granulosa cell isolation

Granulosa cells were isolated from follicular fluid on the day of oocyte retrieval. The cells underwent incubation with hyaluronidase and Hanks’ balanced salt solution, followed by the addition of a human peripheral blood lymphocyte isolate and subsequent centrifugation. The interphase solution was collected, and cell aggregates were removed via filtration. The cells were then transferred to a new microcentrifuge tube using phosphate-buffered saline. After discarding the supernatant, the granulosa cells were prepared for further experimental procedures.

### RNA extraction and quantification

Granulosa cells were subjected to lysis using TRIzol Reagent (Invitrogen) to facilitate total RNA extraction, followed by purification with the RNeasy Mini Kit (Qiagen). The concentration and purity of the isolated total RNA were assessed via ultraviolet spectrophotometry. The extracted RNA was then reverse-transcribed into complementary DNA (cDNA) utilizing the SuperScript II Kit (Invitrogen) and subsequently analyzed through real-time quantitative polymerase chain reaction (qPCR). The mRNA expression levels were normalized to *β-ACTIN* mRNA, which functioned as an internal control, and quantified using the 2^−ΔΔCT^ method. Data are expressed as the mean ± standard error of the mean (SEM) and are based on three independent experiments. The sequences of the PCR primers used were as follows: human *CNOT6L* forward primer AGAAATCTCGGGTAGAGTGCG and reverse primer AGCTTGGCAATATCAGGTGGA; mouse *Cnot6l* forward primer CAGCTGTAGTGGAAAGAATGGAAG and reverse primer CAAGCACGTTCATAT GAGTCTTGG; human *β-ACTIN* forward primer CATGTACGTTGCTATCCAGGC and reverse primer CTCCTTAATGTCACGCACGAT; mouse *β-Actin* forward primer GTGACGTTGACATCCGTAAAGA and reverse primer GCCGGACTCATCGTACT CC.

### Mouse model

Prenatally androgenized mouse model (PNA) were generated as previously described (Liu et al., 2025). In this study, all mice were maintained on a C57BL/6J genetic background and procured from Gempharmatech Co., Ltd. (Nanjing, China). The mice were randomly assigned to experimental groups and provided with *ad libitum* access to a commercially available pelleted diet (Beijing Keao Xieli Feed Co.) and water. The animals were housed under controlled environmental conditions at a temperature of 21°C ± 1°C and a humidity level of 50% ± 5%, with a 12-h light/dark cycle. To establish PNA mouse model using dihydrotestosterone (DHT), pregnant female mice aged 8 weeks were subcutaneously administered DHT at a dose of 250 µg per animal once daily for three consecutive days during embryonic days 16.5–18.5. All female offspring were utilized as PCOS-like model mice and control mice. Ethical approval for all animal studies was obtained from the Animal Care and Use Committee of Guangdong Academy of Medical Sciences (S2022-258-01), and the experiments were conducted in accordance with the guidelines established by the National Institutes of Health for the care and use of animals.

### Cell culture and treatment

At 3 weeks of age, the mice received intraperitoneal injections of pregnant mare serum gonadotropin PMSG at a dose of 5 IU per mouse. Ovarian tissues were collected 24 h post-injection, and follicles were punctured with insulin needles to release granulosa cells into a pre-warmed DMEM/F12 medium. The follicular fluid, enriched with granulosa cells, underwent centrifugation at room temperature, and the resulting cell pellet was resuspended in phosphate-buffered saline (PBS). An equal volume of Ficoll was added for gradient centrifugation, and the intermediate white flocculent layer was carefully aspirated. The cell suspension was then subjected to digestion with hyaluronidase and erythrocyte lysis. Subsequently, the ovarian granulosa cells were seeded onto cell culture dishes or well plates coated with mouse-derived substrate, and adherence was assessed after 24 h. The culture medium was gently replaced, and the subsequent cultured in DMEM/F12 medium with 10% fetal bovine serum, 100 U/ml penicillin and 100ug/ml streptomycin. To investigate the long-term effects of hyperandrogenism on primary cells, these cells were cultured *in vitro* using DMEM-F12 medium supplemented with 10 µM testosterone. We exogenously added 10uM Forskolin to simulate the state of activation of the cAMP signaling pathway.

### Generation of *Cnot6l* overexpressing cells

Recombinant adenoviruses overexpressing *Cnot6l* and *Cnot6* were generated utilizing the recombinant adenovirus system provided by Shanghai Jikai Gene Technology Co., Ltd. Within this system, adenoviral shuttle plasmids containing exogenous genes are co-transfected with packaging plasmids that harbor the adenoviral genome into HEK293 cells. Recombination is facilitated by the Cre/loxP recombinase system, resulting in the production of recombinant adenoviruses. We utilized adenovirus vectors overexpressing *Cnot6l* or *Cnot6* to infect primary mouse ovarian granulosa cells. Upon reaching approximately 70% confluence, the transfection system was quantified based on the cell count and the number of infected replicates. Following a 48-h period post-adenoviral transfection, we conducted cell function assays and various molecular experiments.

### Pyruvate assay and lactate assays

Cellular concentrations of pyruvate and lactate were quantified utilizing a pyruvate colorimetric assay kit and a lactate colorimetric assay kit (Nanjing Jiancheng Bioengineering Institute, China). In summary, cells were harvested and subjected to washing with ice-cold phosphate-buffered saline, followed by lysis and extraction using the buffers provided in the respective kits, adhering strictly to the manufacturer’s protocols. The levels of the metabolites, pyruvate and lactate, were subsequently determined in accordance with the manufacturer’s guidelines.

### ATP and ATPase activity

Cells were seeded into 24-well plates and subjected to various experimental treatments. Following lysis, the cells were centrifuged at 12,000 g for 5 min at 4°C, and the supernatant was collected. Cellular ATP production was quantified using an Enhanced ATP Assay Kit (Beyotime, China). Before ATP measurement, the detection solution was added to a 96-well plate and incubated at room temperature for 5 min. Subsequently, the supernatant was introduced to the plate, rapidly mixed, and analyzed within 30 min. Total ATP levels were derived from luminescence signals and normalized to protein concentrations. The activity of Na^+^-K^+^-ATPase was evaluated by quantifying the release of inorganic phosphate (Pi) from ATP, following the protocol provided by the Nanjing Jiancheng Bioengineering Institute, China. Na^+^-K^+^-ATPase activity was determined using the malachite green dye method to measure the amount of inorganic phosphate, which was expressed as micromoles per milligram of protein.

### Bioenergetic assays

The glycolytic and mitochondrial activity were quantified using a Seahorse XF96 analyzer (Seahorse Bioscience). The Micro BCA assay (Sigma‐Aldrich) determined the cellular protein concentration. The data are presented as ECAR or OCR values normalized to protein concentration.

In the glycolytic activity assay, cells overexpressing *Cnot6l*, along with control cells, were seeded overnight in Seahorse XF96 microplates and incubated in a non-buffered assay medium within a non-CO2 incubator for 30 min prior to the assay. The extracellular acidification rate (ECAR) was recorded over intervals of 4 min, with a mixing duration of 2 min per cycle across four cycles. Following each cycle, activators and inhibitors sourced from Sigma were administered at the following concentrations: glucose, oligomycin, and 2-deoxy-D-glucose.

In the mitochondrial activity assay, cells were seeded into Seahorse miniplates (96 wells) overnight. After which cells were washed and exchanged in Seahorse assay buffer in a 37°C non-CO2 incubator for 30 min before assay, pharmaceutical compounds including oligomycin, FCCP, antimycin, and rotenone were prepared to stressor mix at optimized concentration.

The glycolytic rate is defined as the ECAR achieved by the cell population upon the addition of saturating concentrations of glucose. Glycolytic capacity refers to the maximum ECAR attained by the cell population following the addition of oligomycin, which inhibits oxidative phosphorylation and compels the cells to rely on glycolysis to its fullest extent. The basal OCR is defined as the OCR before the injection of oligomycin. Maximum OCR refers to OCR following the injection of FCCP. Reserve respiration refers to maximum respiration minus basal OCR. Non-mitochondrial OCR refers to the OCR following the injection of rotenone and antimycin A.

### Full-length transcriptome sequencing and analysis

We conducted third-generation RNA sequencing using the PacBio RS II system on Iso-Seq libraries of polyadenylated and full-length transcripts derived from granulosa cells of both PCOS and control groups. The Agilent 2100 Bioanalyzer is employed to assess the concentration, RNA Integrity Number value, 28S/18S ratio, and fragment size of total RNA. The library construction workflow involves several key steps: initially, total RNA samples obtained from granulosa cells of patients with PCOS and control subjects are purified using oligo(dT)-attached magnetic beads. Prior to reverse transcription, the total RNA is linked to a GI anchor to facilitate the reverse transcription of the complete polyadenylated sequence. The RNA, once cleaned and end-extended, is reverse transcribed into full-length cDNA through reverse transcription (Picelli et al., 2014; Jing et al., 2019). The full-length cDNA is amplified using KAPA HiFi HotStart ReadyMix with specific PCR primers. The amplified cDNA products are size-selected using Pure PB beads and subsequently converted into SMRTbell Template libraries using the SMRTbell Template Prep Kit (Liu et al., 2023).

Sequencing data are processed through Reads Of Insert (ROI), full-length transcript classification, cluster and polish to consensus reads. Consensus reads are then mapped to reference genome for funsion gene detectionisoform classification, functional annotation, and coding capacity prediction. Obtained new transcripts with protein coding capacity are added to reference genes for complete reference sequences, followed by gene and isoform expression calculation. Finally, DEGs are detected for multiple samples, and further analysis, such as cluster and functional enrichment analysis are supplied. All sequencing data is available at GEO database (https://www.ncbi.nlm.nih.gov/geo/).

### Statistical analysis

Statistical analyses and graphical representations were performed using SPSS version 29.0 and GraphPad Prism version 9.5. Prior to analysis, normality tests were conducted. Data from the mouse study are presented as mean ± standard error of the mean (SEM) for the specified observations. Comparisons between the two groups were evaluated using either an unpaired two-tailed Student’s t-test or the Mann-Whitney test. For comparisons involving multiple groups, one-way or two-way ANOVA, or a mixed-effects analysis model with multiple comparisons test, was utilized. Detailed statistical information, including sample sizes, is provided in the respective figure legends for each experiment. Statistical significance was determined at a P-value of less than 0.05. Although sample sizes were not predetermined by statistical methods, they align with those reported in previous studies. All experiments requiring statistical analysis were conducted in triplicate, consistently yielding reliable results.

## Results

### *CNOT6L* mRNA levels in the ovaries of PCOS patients and mouse models

**FIGURE 1:**
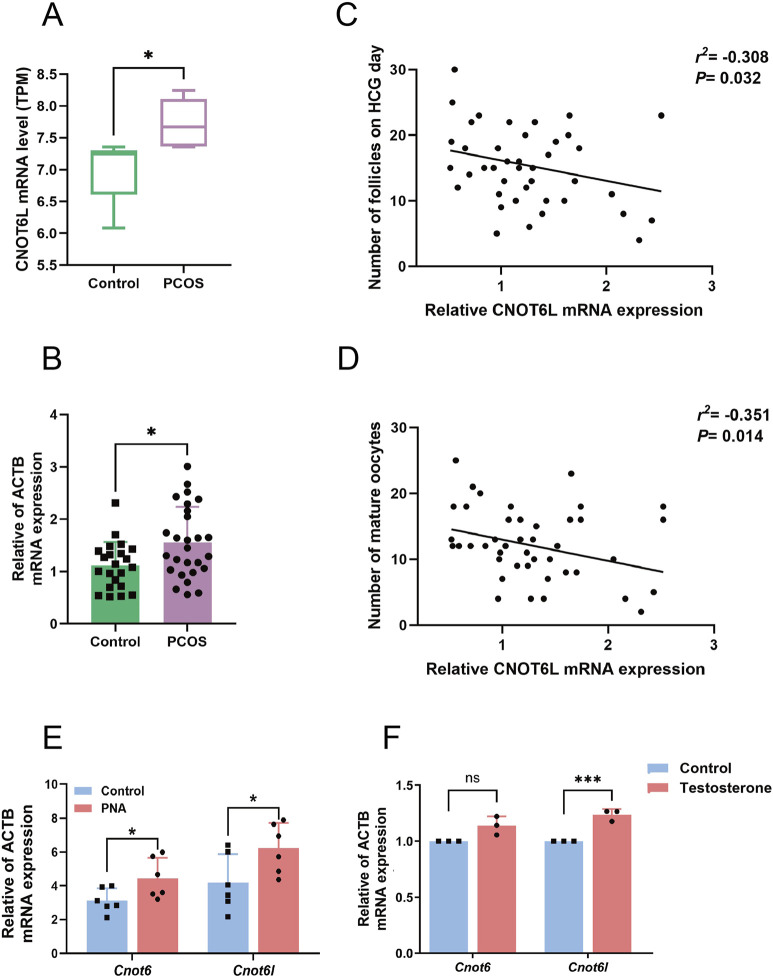
*CNOT6L* mRNA levels in ovarian granulosa cells of PCOS **(A)** *CNOT6L* mRNA expression in ovarian granulosa cells of PCOS patients from public sequencing data. Expression levels were quantified and represented as normalized Transcripts Per Million (TPM) values. **(B)** qPCR analysis of *CNOT6L* in granulosa cells from Control and PCOS patients. n = 22 in Control and n = 27 in PCOS. **(C)** Correlations of *CNOT6L* mRNA expression with the number of follicles on HCG day. n = 49. **(D)** Correlations between the expression of *CNOT6L* mRNA and the number of mature oocytes. n = 49. **(E)** *Cnot6l* and *Cnot6* mRNA expression in control and PNA mouse ovaries. n = 5 in Control and n = 6 in PNA. **(F)** *Cnot6l* and *Cnot6* mRNA expression in granulosa cells treated with testosterone. *P < 0.05, **P < 0.01, and ***P < 0.001.

By re-analyzing previously reported sequencing data of ovarian granulosa cells in polycystic ovary syndrome, which were GSE138518, GSE155489, and GSE173160, we identified a significant increase in *CNOT6L* mRNA expression levels in the ovarian granulosa cells of individuals with PCOS (Figure 1A). Subsequently, we collected granulosa cell samples from PCOS patients undergoing IVF-ET at the reproductive medicine center, as well as from a control group, to validate these findings. The results confirmed elevated *CNOT6L* mRNA expression levels in the granulosa cells of PCOS patients compared to those in the control group (Figure 1B). Furthermore, the mRNA expression level of *CNOT6L* in the ovarian granulosa cells of PCOS patients exhibited correlations with the patients’ body mass index, the number of follicles on hCG day, and the number of mature oocytes. Although these correlations were relatively weak, they were statistically significant. Specifically, the mRNA expression of *CNOT6L* demonstrated a weak positive correlation with BMI and weak negative correlations with the number of follicles on hCG day and the number of mature oocytes, with −0.308 and −0.351 correlation coefficients, respectively (Figures 1C,D).

Subsequently, we established a mouse model of PCOS through the administration of dihydrotestosterone during the late gestational period. We analyzed the mRNA expression levels in the ovaries of mice, comparing those with PCOS to control subjects. The findings indicated a significant upregulation of the transcriptional levels of key genes involved in mRNA deadenylation in the ovarian tissues of PCOS model mice compared to the control group (Figure 1E). Following this, we cultured ovarian granulosa cells from wild-type mice *in vitro* and administered exogenous testosterone stimulation. The results indicate that the mRNA expression level of *Cnot6l* in ovarian granulosa cells increased following testosterone treatment. The expression level of *Cnot6* exhibited only a mild elevation, but the difference did not reach statistical significance (Figure 1F). These results suggest that the expression of the key gene CNOT6L, which is implicated in the regulation of mRNA deadenylation, is altered in the ovarian granulosa cells of both PCOS patients and animal models.

### Overexpressing CNOT6L affects mouse ovarian granulosa cell function

**FIGURE 2:**
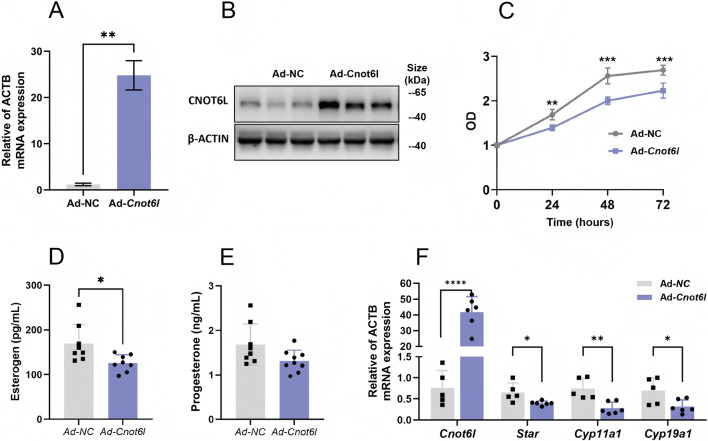
Effects of CNOT6L-overexpression on ovarian granulosa cell function. **(A,B)** the effect of CNOT6L-overexpression on its mRNA levels and protein levels in primary granulosa cells. **(C)** Cell proliferation ability was detected by CCK-8 assay in *Cnot6l*-overexpression cells. **(D,E)** 17β-Esterogen and Progesterone levels in cell medium. **(F)** qRT-PCR analysis of hormone related genes in *Cnot6l*-overexpress granulosa cells. *P < 0.05, **P < 0.01, and ***P < 0.001.

We engineered adenoviruses to overexpress *Cnot6* and *Cnot6l* and subsequently transfected primary granulosa cells derived from wild-type mice with these constructs. Following transfection, we quantitatively analyzed the mRNA and protein expression levels of CNOT6L in the granulosa cells exhibiting overexpression. Our findings indicated a significant upregulation of both mRNA and protein levels of CNOT6L in the overexpression group compared to cells transfected with an empty vector adenovirus (Figures 2A,B). Furthermore, we evaluated the proliferative capacity of the cells, observing a marked reduction in the proliferative ability of *Cnot6l* overexpressing cells at 24 h post-transfection, with the most pronounced difference occurring at 48 h (Figure 2C). We assessed hormone concentrations in the cell culture medium, revealing that the aberrant overexpression of CNOT6L in granulosa cells resulted in a statistically significant reduction in estrogen levels (Figure 2D). In contrast, progesterone levels exhibited a minor decrease that did not reach statistical significance (Figure 2E). Additionally, we investigated the expression of genes associated with steroid hormone synthesis within the cells. Our analysis demonstrated a significant downregulation of mRNA levels for critical enzymes involved in steroid hormone synthesis in granulosa cells overexpressing CNOT6L (Figure 2F).

### Overexpressing CNOT6L disrupts energy metabolism in ovarian granulosa cells

**FIGURE 3:**
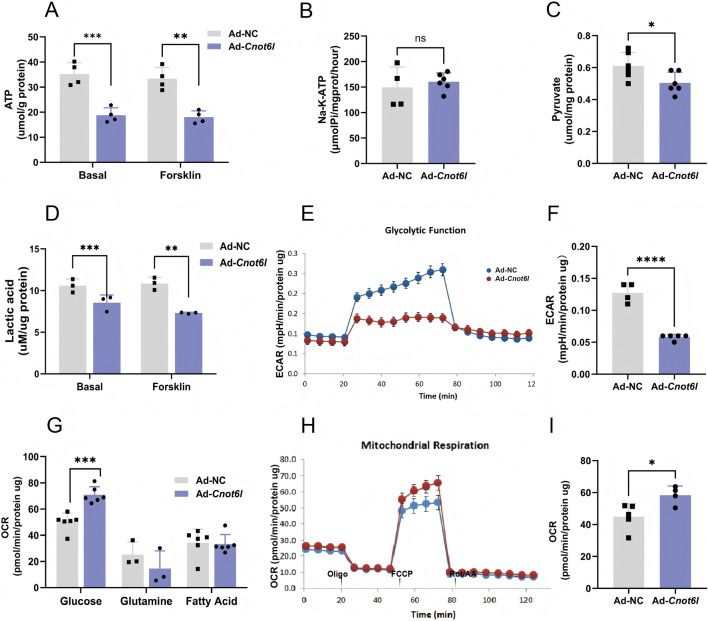
CNOT6L overexpression disrupts energy metabolism in granulosa cells. **(A)** Intracellular changes of ATP. **(B)** The activity of Na-K-ATPase in granulosa cells. **(C)** Intracellular level of pyruvate. **(D)** Intracellular lactic acid levels under various treatments. **(E)** Real-time monitoring of critical glycolytic parameters utilizing the Seahorse XFe96 analyzer. **(F)** Monitoring key glycolytic parameters in real-time enables calculation of maximum glycolytic capacity. **(G)** Results of Mitochondrial ATP inhibition substrate test. **(H)** Real-time monitoring oxygen consumption rate (OCR) of mitochondrial stress test utilizing the Seahorse XFe96 analyzer. **(I)** Monitoring oxygen consumption rate parameters in real-time enables calculation of maximal respiration. *P < 0.05, **P < 0.01, and ***P < 0.001.

Previous studies have indicated that the glycolytic processes and mitochondrial function in ovarian granulosa cells of patients with polycystic ovary syndrome are compromised. This impairment leads to damage in the granulosa cells, which subsequently diminishes oocyte quality and adversely affects embryonic developmental potential (Zhang et al., 2022; Cao et al., 2022). Oocytes predominantly depend on the direct utilization of pyruvate to fulfill their energy requirements during maturation. Given the critical role of energy metabolism homeostasis in granulosa cells for follicular development, we further assessed the energy metabolism status in *Cnot6l* overexpressing cells. The results demonstrated that overexpression of CNOT6L significantly reduced ATP levels in granulosa cells under both basal and cAMP-activated conditions (Figure 3A). However, overexpression of CNOT6L did not significantly alter the activity of Na-K-ATPase (Figure 3B). Moreover, the levels of lactate and pyruvate, which serve as energy substrates for oocytes, were significantly decreased (Figures 3C,D). Subsequently, a cellular energy metabolism analyzer was employed to evaluate the glycolytic process within the cytoplasm of granulosa cells. The findings indicated a significant inhibition of glycolytic capacity in cells overexpressing CNOT6L (Figure 3E), accompanied by a reduction in maximum glycolytic capacity (Figure 3F). Analysis of primary mitochondrial energy substrates demonstrated a marked preference for glucose-pyruvate-derived sources (Figure 3G). Consequently, mitochondrial oxidative respiration was further assessed in *Cnot6l*-overexpressing cells. The results revealed that CNOT6L overexpression not only diminished glycolytic activity in granulosa cells but also significantly enhanced mitochondrial oxidative respiration (Figure 3H), as evidenced by a substantial increase in the maximum oxygen consumption rate (Figure 3I). Collectively, these findings confirm that modulating the levels of the deadenylase CNOT6L in ovarian granulosa cells can shift intracellular energy utilization from glycolysis to mitochondrial oxidative phosphorylation. This alteration subsequently hinders the transport of energy substrates from granulosa cells to oocytes, resulting in energy depletion within the oocytes.

### Transcriptome sequencing of granulosa cells from PCOS patients

The stability and degradation of mRNAs are correlated with the lengths of their poly(A) tails. Drawing upon previous research findings, we propose the hypothesis that variations in the levels of the deadenylase enzyme CNOT6L in ovarian granulosa cells may influence follicular developmental dysfunction in polycystic ovary syndrome by modulating energy metabolism pathways. To explore the pathways involved in greater depth, we collected granulosa cells from patients diagnosed with PCOS as well as from control subjects. Samples were utilized to construct Iso-Seq libraries, which were subsequently employed for full-length transcript sequencing using the PacBio RS II system. This approach was implemented to evaluate RNA poly(A) tail lengths.

**FIGURE 4:**
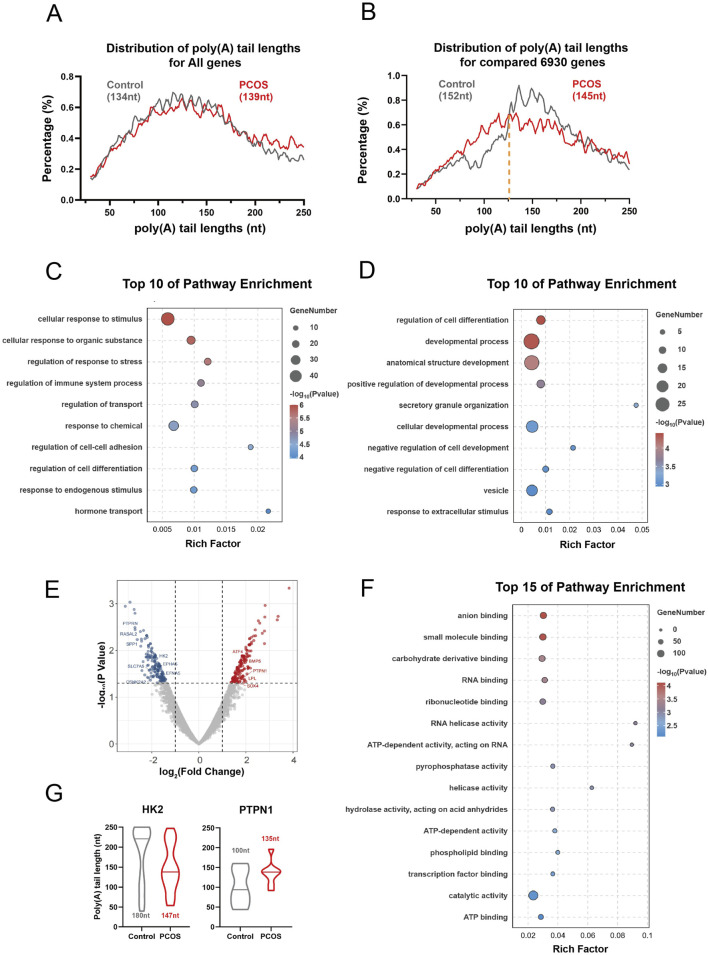
Full-length transcriptome sequencing for granulosa cells. **(A)** ISO-seq assay showing distribution of poly(A) tail lengths for all genes in control and PCOS granulosa cells. The median poly(A) tail lengths for all genes of group were presented in parentheses. **(B)** The ISO-seq assay displays poly(A) tail length distribution for pairwise genes in control and PCOS granulosa cells, with median lengths for each group in parentheses and yellow dotted line marking the distribution shift. nt, nucleotide. **(C,D)** GO enrichment analysis for the pairwise genes according to the mean lengths of poly(A) tails. **(E)** The volcano plots of differentially expressed genes. **(F)** The GO analysis of differentially expressed genes. **(G)** The length of the Poly(A) tail in the differentially expressed genes *HK2* and *PTPN1*.

In all cells, 13,473 genes (61,093 transcripts) and 13,966 genes (62,072 transcripts) were captured from the Control and PCOS groups, respectively. The transcripts in the duplicates exhibited a comparable distribution of poly(A) tail lengths. Notably, for mRNAs encoded by the nuclear genome, the median poly(A) tail lengths in PCOS cells (139 nucleotides) were similar to those in control cells (134 nucleotides) (Figure 4A). However, a pairwise comparison of the genes (n = 6,930) that were concurrently expressed in both PCOS and control cells revealed an increase in the fraction of transcripts with poly(A) tails ranging from 30 to 126 nucleotides in PCOS cells compared to the Control group. Conversely, the proportion of transcripts with poly(A) tails measuring 126 to 250 nucleotides was reduced in PCOS cells relative to the Control group (Figure 4B). To elucidate the functional implications of genes with altered polyadenylation tails, we performed a Gene Ontology (GO) enrichment analysis on genes displaying either elongated or truncated poly(A) tails in the PCOS group relative to the control group. The GO analysis indicated that genes associated with immune regulation, response to stimuli, and cell adhesion predominantly exhibited elongated poly(A) tails (Figure 4C). In contrast, genes involved in cell differentiation, development, and vesicle transport processes were characterized by truncated poly(A) tails in the PCOS group (Figure 4D). Subsequent analysis of the differentially expressed genes within the same samples was conducted. Differentially expressed genes were defined based on a P-value threshold of <0.05 and an absolute fold change of >1.5. The results indicated a total of 191 downregulated genes and 181 upregulated genes in the PCOS group in comparedison to the control group (Figure 4E). GO enrichment analysis of differentially expressed genes demonstrated a significant enrichment in pathways associated with anion binding, small molecule binding, and carbohydrate derivative binding. Importantly, pathways pertinent to energy production and utilization, including ATP-dependent activity and ATP binding, were also significantly enriched in the polycystic ovary syndrome group (Figure 4F). By correlating differentially expressed genes with their respective transcripts, we identified 93 genes that not only demonstrated differential expression but also exhibited variations in polyadenylation tail length. Among these, 44 genes were upregulated, whereas 49 genes were downregulated in the PCOS group. The genes in question include several that play pivotal roles in metabolic processes, notably the HK2 gene, which exhibits reduced expression levels and a shortened polyadenylation tail in individuals with polycystic ovary syndrome (Figure 4G). Furthermore, the analysis identifies critical genes involved in gamete development, such as the PTPN1 gene, which shows increased expression and an elongated polyadenylation tail in the PCOS group compared to the control group (Figure 4G).

## Discussion

In patients with PCOS, abnormal proliferation and dysfunction of granulosa cells in early-developing follicles impede normal oocyte development (Franks et al., 2008). Ovarian granulosa cells generate pyruvate, lactate, and other metabolic intermediates essential for oocyte development. Disruptions in granulosa cell glycolysis and aberrant pyruvate levels have been associated with the emergence of reproductive endocrine disorders (Huang et al., 2022). Studies have shown that glucose utilization by the ovaries in rat models of PCOS is markedly reduced, resulting in diminished glucose accumulation and lactate production in these models (Tanaka et al., 2021). The inhibition of mitochondrial pyruvate uptake in ovarian organoid tissues has been demonstrated to suppress early folliculogenesis, thereby impairing subsequent follicular development (Mazloomi et al., 2023). Granulosa cells in PCOS patients exhibit disrupted energy regulation, with reduced glycolysis resulting in lower pyruvate and lactate levels essential for oocyte development (Huo et al., 2023). PCOS is also characterized by increased mitochondrial activity, elevated reactive oxygen species, and diminished antioxidant capacity (Lai et al., 2018). The resultant decrease in energy, attributed to impaired glycolysis and mitochondrial dysfunction in granulosa cells, is associated with the presence of immature oocytes and the occurrence of biochemical pregnancy loss (Mazloomi et al., 2023). Prior studies have substantiated that promoting the transition from glycolysis to the pentose phosphate pathway impedes mitochondrial oxidative phosphorylation in granulosa cells, consequently affecting the energy metabolism homeostasis within the follicular microenvironment (Zhao et al., 2023). Our study mainly examines the glycolysis and mitochondrial respiration in granulosa cells. We propose a scientifically plausible hypothesis: mitochondrial dysfunction and aberrant pyruvate metabolism within granulosa cells may lead to insufficient energy provision to oocytes, potentially impairing follicular development in PCOS. Examining the factors that disturb the energy metabolism homeostasis in granulosa cells could offer a novel perspective for understanding the pathogenesis of PCOS.

The growth of follicles and the normal development of oocytes depend on adequate energy, cytokines, and hormones, all supported by granulosa cells’ proliferation and endocrine functions. Previous studies have suggested that FSH stimulates the proliferation of ovarian granulosa cells by activating the transcription of key hormone synthesis genes in granulosa cells, promoting the synthesis of steroid hormones in growing follicles, and preventing follicular atresia. Dai et al. proposed that the deadenylase *Cnot6*/*6l*, identified as a novel target gene of follicle-stimulating hormone, facilitates deadenylation to downregulate developmental inhibitors such as anti-Mullerian hormone in ovarian follicles (Dai et al., 2021). These findings provide new insights into the hypothesis that post-transcriptional regulation in granulosa cells plays a role in follicular development. Building on these findings, we observed increased expression levels of CNOT6L in ovarian granulosa cells from both PCOS patients and animal models. Furthermore, our study revealed that CNOT6L overexpression inhibits the maximal glycolytic capacity in granulosa cells while simultaneously enhancing the maximal respiratory rate, intermediate metabolites such as pyruvate and lactate were reduced in granulosa cells overexpressing CNOT6L, indicating a disruption in energy metabolic homeostasis. These results are consistent with previous research conducted on clinical samples from PCOS patients. This implies that the excessive activation of CNOT6L in granulosa cells, induced by various factors, may mediate metabolic disturbances through the process of deadenylation and could be associated with the disordered follicle development observed in polycystic ovary syndrome.

In this study, we conducted full-length transcriptome sequencing of granulosa cells derived from patients with polycystic ovary syndrome and performed a preliminary functional analysis of genes and transcripts that exhibited variations in polyadenylation tail length between the PCOS and control groups. The results demonstrated a notable enrichment in pathways related to cellular responses to stimuli, regulation of immune system processes, regulation of cell differentiation, cellular developmental processes, and hormone transport. Additionally, we identified differentially expressed genes associated with variations in the polyadenylation tail length of transcripts. These genes are closely associated with energy metabolism processes, including hexokinase-2 (*HK2*) and protein tyrosine phosphatase non-receptor type 1 (*PTPN1*). Previous research has demonstrated that the inadequate energy production associated with glycolysis in granulosa cells plays a crucial role in the development of follicular dysplasia in polycystic ovary syndrome. The variable expression of exosomal microRNAs in follicular fluid, notably miR-143-3p and miR-155-5p, modulates the glycolytic pathway in granulosa cells through the regulation of HK2. This modulation affects follicular development in PCOS by altering ATP synthesis, lactate levels, and apoptotic processes (Cao et al., 2022). In our study, we observed that the reduction in HK2 transcriptional levels in granulosa cells of the PCOS group is linked to the shortening of the poly(A) tail length of its transcript. Furthermore, we concentrated on PTPN1 due to the substantial body of research indicating that PTPN1 variants are associated with obesity, diabetes, and insulin resistance (Bento et al., 2004). PTPN1 is considered a critical molecule in mediating insulin resistance and obesity in females. Female mice with a PTPN1 knockout exhibit phenotypes such as enhanced insulin sensitivity and a significant reduction in body fat content (Tsou and Bence, 2012; Kerbus et al., 2023). In our investigations, the poly(A) tail of the PTPN1 transcript was significantly longer than that of the control group, suggesting a greater propensity for accumulation.

The sequencing results revealed differential expression of several key genes related to energy metabolism. However, alterations in poly(A) tail length were not statistically significant, as exemplified by the case of lactate dehydrogenase A (LDHA). These findings imply that mRNA deadenylation, mediated by factors such as CNOT6L, plays a crucial role in the regulation of energy metabolism in granulosa cells. Furthermore, multiple epigenetic regulatory mechanisms are implicated in this process. While this study has not yet pinpointed the specific molecule responsible for the upregulation of Cnot6l expression in ovarian granulosa cells associated with polycystic ovary syndrome, it offers valuable insights for future research employing PCOS animal models to explore the mechanisms underlying follicular developmental disorders. Furthermore, the identification and validation of molecular interactions between CNOT6L and genes such as HK2 will constitute a primary objective in the subsequent phase of our research.

## Conclusion

Ovarian follicular developmental disorders and disruptions in energy homeostasis are principal pathological features observed in the ovaries of individuals with PCOS. The mRNA deadenylase CNOT6L is significantly upregulated in the ovarian granulosa cells of both PCOS patients and corresponding mouse models. CNOT6L plays a crucial role in the precise regulation of oocyte maturation processes and energy homeostasis across peripheral tissues and organs. The overexpression of CNOT6L inhibits the glycolytic pathway while activating the mitochondrial oxidative phosphorylation pathway, leading to reduced lactate production and consequently impairing the energy supply to the oocyte. This study examines the effects of aberrantly elevated CNOT6L levels and variations in mRNA poly(A) tail length on granulosa cell dysfunction in PCOS. The findings provide novel insights into the role of CNOT6L in regulating energy metabolism homeostasis and its involvement in follicular developmental disorders associated with polycystic ovary syndrome.

## Data Availability

The datasets presented in this study can be found in online repositories. The names of the repository/repositories and accession number(s) can be found below: https://www.ncbi.nlm.nih.gov/geo/query/acc.cgi?acc=GSE294074.

## References

[B1] Babayev E. Duncan F. E. (2022). Age-associated changes in cumulus cells and follicular fluid: the local oocyte microenvironment as a determinant of gamete quality. Biol. Reprod. 106 (2), 351–365. 10.1093/biolre/ioab241 34982142 PMC8862720

[B2] Bento J. L. Palmer N. D. Mychaleckyj J. C. Lange L. A. Langefeld C. D. Rich S. S. (2004). Association of protein tyrosine phosphatase 1B gene polymorphisms with type 2 diabetes. Diabetes 53 (11), 3007–3012. 10.2337/diabetes.53.11.3007 15504984

[B3] Brachova P. Alvarez N. S. Christenson L. K. (2021). Loss of cnot6l impairs inosine RNA modifications in mouse oocytes. Int. J. Mol. Sci. 22 (3), 1191. 10.3390/ijms22031191 33530472 PMC7865253

[B4] Cao J. Huo P. Cui K. Wei H. Cao J. Wang J. (2022). Follicular fluid-derived exosomal miR-143-3p/miR-155-5p regulate follicular dysplasia by modulating glycolysis in granulosa cells in polycystic ovary syndrome. Cell Commun. Signal 20 (1), 61. 10.1186/s12964-022-00876-6 35534864 PMC9082924

[B5] Dai X. Zhi-Yan J. Yun-Wen W. Sha Q.-Q. Liu Y. Jia-Yi D. (2021). CNOT6/6L-mediated mRNA degradation in ovarian granulosa cells is a key mechanism of gonadotropin-triggered follicle development. Cell Rep. 37 (7), 110007. 10.1016/j.celrep.2021.110007 34788619

[B6] Dai X. X. Pi S. B. Zhao L. W. Wu Y. W. Shen J. L. Zhang S. Y. (2022). PABPN1 functions as a hub in the assembly of nuclear poly(A) domains that are essential for mouse oocyte development. Sci. Adv. 8 (43), n9016. 10.1126/sciadv.abn9016 PMC9616507 36306357

[B7] Escobar-Morreale H. F. (2018). Polycystic ovary syndrome: definition, aetiology, diagnosis and treatment. Nat. Rev. Endocrinol. 14 (5), 270–284. 10.1038/nrendo.2018.24 29569621

[B8] Franks S. Stark J. Hardy K. (2008). Follicle dynamics and anovulation in polycystic ovary syndrome. Hum. Reprod. Update 14 (4), 367–378. 10.1093/humupd/dmn015 18499708

[B9] Huang L. Liang A. Li T. Lei X. Chen X. Liao B. (2022). Mogroside v improves follicular development and ovulation in Young-Adult PCOS rats induced by letrozole and High-Fat diet through promoting glycolysis. Front. Endocrinol. (Lausanne) 13, 838204. 10.3389/fendo.2022.838204 35418943 PMC8995474

[B10] Huo P. Li M. Le J. Zhu C. Yao J. Zhang S. (2023). Resveratrol improves follicular development of PCOS rats via regulating glycolysis pathway and targeting SIRT1. Syst. Biol. Reprod. Med. 69 (2), 153–165. 10.1080/19396368.2022.2125855 36268996

[B11] Jing Y. Zhang Y. Zhu H. Zhang K. Cai M. C. Ma P. (2019). Hybrid sequencing-based personal full-length transcriptomic analysis implicates proteostatic stress in metastatic ovarian cancer. Oncogene 38 (16), 3047–3060. 10.1038/s41388-018-0644-y 30617306

[B12] Joham A. E. Norman R. J. Stener-Victorin E. Legro R. S. Franks S. Moran L. J. (2022). Polycystic ovary syndrome. Lancet Diabetes Endocrinol. 10 (9), 668–680. 10.1016/S2213-8587(22)00163-2 35934017

[B13] Kataoka J. Larsson I. Bjorkman S. Eliasson B. Schmidt J. Stener-Victorin E. (2019). Prevalence of polycystic ovary syndrome in women with severe obesity - effects of a structured weight loss programme. Clin. Endocrinol. (Oxf) 91 (6), 750–758. 10.1111/cen.14098 31529511

[B14] Katsumura S. Siddiqui N. Goldsmith M. R. Cheah J. H. Fujikawa T. Minegishi G. (2022). Deadenylase-dependent mRNA decay of GDF15 and FGF21 orchestrates food intake and energy expenditure. Cell Metab. 34 (4), 564–580.e8. 10.1016/j.cmet.2022.03.005 35385705 PMC9386786

[B15] Kerbus R. I. Inglis M. A. Anderson G. M. (2023). Neuronal Ptpn1 and Socs3 deletion improves metabolism but not anovulation in a mouse polycystic ovary syndrome model. J. Endocrinol. 259 (1), e230023. 10.1530/JOE-23-0023 37466473

[B16] Lai Q. Xiang W. Li Q. Zhang H. Li Y. Zhu G. (2018). Oxidative stress in granulosa cells contributes to poor oocyte quality and IVF-ET outcomes in women with polycystic ovary syndrome. Front. Med. 12 (5), 518–524. 10.1007/s11684-017-0575-y 29260383

[B17] Li J. Chen H. Gou M. Tian C. Wang H. Song X. (2021). Molecular features of polycystic ovary syndrome revealed by transcriptome analysis of oocytes and cumulus cells. Front. Cell Dev. Biol. 9, 735684. 10.3389/fcell.2021.735684 34552933 PMC8450412

[B18] Liu Y. Dong Y. Jiang Y. Han S. Liu X. Xu X. (2025). Caloric restriction prevents inheritance of polycystic ovary syndrome through oocyte-mediated DNA methylation reprogramming. Cell Metab. 37 (4), 920–935.e6. 10.1016/j.cmet.2025.01.014 39986273

[B19] Liu Y. Nie H. Zhang Y. Lu F. Wang J. (2023). Comprehensive analysis of mRNA poly(A) tails by PAIso-seq2. Sci. China Life Sci. 66 (1), 187–190. 10.1007/s11427-022-2186-8 36044132

[B20] Mazloomi S. Farimani M. S. Tavilani H. Karimi J. Amiri I. Abbasi E. (2023). Granulosa cells from immature follicles exhibit restricted glycolysis and reduced energy production: a dominant problem in polycystic ovary syndrome. J. Assist. Reprod. Genet. 40 (2), 343–359. 10.1007/s10815-022-02676-w 36593322 PMC9935788

[B21] Mimouni N. Paiva I. Barbotin A. L. Timzoura F. E. Plassard D. Le Gras S. (2021). Polycystic ovary syndrome is transmitted via a transgenerational epigenetic process. Cell Metab. 33 (3), 513–530.e8. 10.1016/j.cmet.2021.01.004 33539777 PMC7928942

[B22] Neven A. Laven J. Teede H. J. Boyle J. A. (2018). A summary on polycystic ovary syndrome: diagnostic criteria, prevalence, clinical manifestations, and management according to the latest international guidelines. Semin. Reprod. Med. 36 (1), 5–12. 10.1055/s-0038-1668085 30189445

[B23] Picelli S. Faridani O. R. Bjorklund A. K. Winberg G. Sagasser S. Sandberg R. (2014). Full-length RNA-seq from single cells using Smart-seq2. Nat. Protoc. 9 (1), 171–181. 10.1038/nprot.2014.006 24385147

[B24] Qiao J. Feng H. L. (2011). Extra- and intra-ovarian factors in polycystic ovary syndrome: impact on oocyte maturation and embryo developmental competence. Hum. Reprod. Update 17 (1), 17–33. 10.1093/humupd/dmq032 20639519 PMC3001338

[B25] Tanaka K. Hayashi Y. Takehara A. Ito-Matsuoka Y. Tachibana M. Yaegashi N. (2021). Abnormal early folliculogenesis due to impeded pyruvate metabolism in mouse oocytes†. Biol. Reprod. 105 (1), 64–75. 10.1093/biolre/ioab064 33824958

[B26] Tsou R. C. Bence K. K. (2012). The genetics of PTPN1 and obesity: insights from mouse models of Tissue-Specific PTP1B deficiency. J. Obes. 2012, 926857. 10.1155/2012/926857 22811891 PMC3395189

[B27] Xu S. Zhang Y. Qiang C. Zhang C. (2022). Effect of TSH on oocyte maturation of PCOS patients with normal thyroid function in IVF. Reprod. Biol. Endocrinol. 20 (1), 133. 10.1186/s12958-022-01005-1 36056438 PMC9438297

[B28] Yerushalmi G. M. Salmon-Divon M. Yung Y. Maman E. Kedem A. Ophir L. (2014). Characterization of the human cumulus cell transcriptome during final follicular maturation and ovulation. Mol. Hum. Reprod. 20 (8), 719–735. 10.1093/molehr/gau031 24770949

[B29] Yildiz B. O. Bozdag G. Yapici Z. Esinler I. Yarali H. (2012). Prevalence, phenotype and cardiometabolic risk of polycystic ovary syndrome under different diagnostic criteria. Hum. Reprod. 27 (10), 3067–3073. 10.1093/humrep/des232 22777527

[B30] Zhang X. Zhang W. Wang Z. Zheng N. Yuan F. Li B. (2022). Enhanced glycolysis in granulosa cells promotes the activation of primordial follicles through mTOR signaling. Cell Death Dis. 13 (1), 87. 10.1038/s41419-022-04541-1 35087042 PMC8795455

[B31] Zhang Y. Liu G. Ding H. Fan B. (2024). High expression of CNOT6L contributes to the negative development of type 2 diabetes. Sci. Rep. 14 (1), 24723. 10.1038/s41598-024-76095-5 39433858 PMC11494123

[B32] Zhao Y. K. Gao Y. N. Wang L. C. Wang J. Wang G. J. Wu H. L. (2023). Correlation between abnormal energy metabolism of ovarian granulosa cells and *in vitro* fertilization-embryo transfer outcomes in patients with polycystic ovary syndrome and obesity. J. Ovarian Res. 16 (1), 145. 10.1186/s13048-023-01204-3 37480140 PMC10362761

